# Tracking Antimicrobial Resistance Determinants in Diarrheal Pathogens: A Cross-Institutional Pilot Study

**DOI:** 10.3390/ijms21165928

**Published:** 2020-08-18

**Authors:** Chris R. Taitt, Tomasz A. Leski, Michael G. Prouty, Gavin W. Ford, Vireak Heang, Brent L. House, Samuel Y. Levin, Jennifer A. Curry, Adel Mansour, Hanan El Mohammady, Momtaz Wasfy, Drake Hamilton Tilley, Michael J. Gregory, Matthew R. Kasper, James Regeimbal, Paul Rios, Guillermo Pimentel, Brook A. Danboise, Christine E. Hulseberg, Elizabeth A. Odundo, Abigael N. Ombogo, Erick K. Cheruiyot, Cliff O. Philip, Gary J. Vora

**Affiliations:** 1 US Naval Research Laboratory, Center for Biomolecular Science & Engineering, Washington, DC 20375, USA; tomasz.leski@nrl.navy.mil (T.A.L.); gary.vora@nrl.navy.mil (G.J.V.); 2 US Naval Medical Research Unit No. 2-Phnom Penh, Blvd Kim Il Sung, Khan Toul Kork, Phnom Penh, Cambodia; michael.g.prouty2.mil@mail.mil (M.G.P.); gavin.w.ford.mil@mail.mil (G.W.F.); vireak.heang.fn@namru2.org.kh (V.H.); 3 US Naval Medical Research Unit No. 3, Naval Air Station Sigonella, 95030 Sigonella, Italy; blhouse@interpathlab.com (B.L.H.); Samuel.y.levin.mil@mail.mil (S.Y.L.); jennifer.a.curry.mil@mail.mil (J.A.C.); adelmansour@hotmail.com (A.M.); hismail@camris.com (H.E.M.); bdd@me-vac.com (M.W.); 4 US Naval Medical Research Unit No. 6 Peru, Lima 07001, Peru; drake.tilley@med.navy.mil (D.H.T.); Michael.j.gregory64.mil@mail.mil (M.J.G.); matthew.r.kasper2.mil@mail.mil (M.R.K.); james.m.regeimbal.mil@mail.mil (J.R.); paul.a.rios12.ln@mail.mil (P.R.); guillermo.pimentel2.mil@mail.mil (G.P.); 5 US Army Medical Research Directorate-Africa/Kenya, Kericho 20200, Kenya; bdanboise@gmail.com (B.A.D.); Christine.e.hulseberg.mil@mail.mil (C.E.H.); Elizabeth.odundo@usamru-k.org (E.A.O.); abigael.ombogo@usamru-k.org (A.N.O.); erick.kipkirui@usamru-k.org (E.K.C.); cliff.philip@usamru-k.org (C.O.P.)

**Keywords:** diarrheal pathogen, antimicrobial resistance, *Campylobacter*, *Shigella*, *Escherichia coli*, *Salmonella*, microarray

## Abstract

Infectious diarrhea affects over four billion individuals annually and causes over a million deaths each year. Though not typically prescribed for treatment of uncomplicated diarrheal disease, antimicrobials serve as a critical part of the armamentarium used to treat severe or persistent cases. Due to widespread over- and misuse of antimicrobials, there has been an alarming increase in global resistance, for which a standardized methodology for geographic surveillance would be highly beneficial. To demonstrate that a standardized methodology could be used to provide molecular surveillance of antimicrobial resistance (AMR) genes, we initiated a pilot study to test 130 diarrheal pathogens (*Campylobacter* spp., *Escherichia coli*, *Salmonella*, and *Shigella* spp.) from the USA, Peru, Egypt, Cambodia, and Kenya for the presence/absence of over 200 AMR determinants. We detected a total of 55 different determinants conferring resistance to ten different categories of antimicrobials: genes detected in ≥ 25 samples included *bla*_TEM_, *tet*(A), *tet*(B), *mac*(A), *mac*(B), *aadA1/A2*, *strA*, *strB*, *sul1*, *sul2*, *qacE*Δ1, *cmr*, and *dfrA1*. The number of determinants per strain ranged from none (several *Campylobacter* spp. strains) to sixteen, with isolates from Egypt harboring a wider variety and greater number of genes per isolate than other sites. Two samples harbored carbapenemase genes, *bla*_OXA-48_ or *bla*_NDM_. Genes conferring resistance to azithromycin (*ere*(A), *mph*(A)/*mph*(K), *erm*(B)), a first-line therapeutic for severe diarrhea, were detected in over 10% of all Enterobacteriaceae tested: these included >25% of the Enterobacteriaceae from Egypt and Kenya. Forty-six percent of the Egyptian Enterobacteriaceae harbored genes encoding CTX-M-1 or CTX-M-9 families of extended-spectrum β-lactamases. Overall, the data provide cross-comparable resistome information to establish regional trends in support of international surveillance activities and potentially guide geospatially informed medical care.

## 1. Introduction

Diarrheal disease ranks among the top 10 leading causes of death for all ages and fifth for children under five years of age, affecting over four billion individuals globally each year in 2016 and 2017 [1,2,3]. Low-income countries are still disproportionately affected by infectious diarrhea [1,4], likely due to poor access to safe drinking water, sanitation, and healthcare [3,5,6].

Many cases of intestinal disease are self-limiting and can be effectively treated with oral rehydration solutions and antisecretory/antimotility drugs [7,8]. While antimicrobial use is not generally recommended as a standard treatment, it is, however, indicated for some clinically recognizable severe or persistent cases (e.g., cholera, dysenteric shigellosis), in immunocompromised individuals or those with underlying risk factors [7,8,9], and to reduce the severity and duration of travelers’ diarrhea [10]. In spite of recommendations for limited use, up to 40% of children under five with diarrhea and up to 80% of persons with acute diarrhea are treated with antimicrobials, regardless of presentation or cause [6,11,12].

Veterinary and aquaculture use is also responsible for a large proportion of global antimicrobial consumption [13,14,15,16,17,18]. For terrestrial animal farming, antimicrobials are used as both prophylactics and growth promoters, whereas they are used in aquaculture to both treat and avoid opportunistic infections of farmed species due to crowded, stressful, or suboptimal culture conditions [18]. To mitigate the potential for animal-to-human transmission of resistance to clinically relevant drugs, many developed countries have restricted antimicrobial use in veterinary and aquacultural practices; however, many low- and middle-income nations lack such national policies or the ability to enforce them [15,19]. Furthermore, though the aquaculture industry has made great strides in antimicrobial stewardship and limiting the number of compounds used, drug spillover from aquacultural use has the potential to affect multiple ecosystems [16,17,20] whereas spillover of drugs from terrestrial animal husbandry is more limited to soil ecosystems. Regardless of the original source of selective pressure for development of drug resistance—human, livestock, or aquaculture—there exists an interplay between multiple ecosystems to disseminate and exchange antimicrobial resistance determinants (ARDs) [16,21,22]. For this reason, a unified, multifaceted approach (e.g., One Health) is needed in order to address both the causes and sequelae of antimicrobial resistance (AMR) [16,23].

Pertinent to the current study, there has been an alarming increase in resistance to drugs commonly used to treat diarrheal illness throughout the world [6,10,24]. This AMR is associated with increased morbidity, mortality, and healthcare costs and has resulted in significant reductions in gross domestic product in many nations [2,25,26].

Compounding the problem of global AMR are geographical differences in pathogen and resistance profiles. Therefore, selecting an appropriate and effective therapeutic strategy for infectious diarrhea—when needed—requires situational awareness of the prevalence and types of AMR pathogens and mechanisms for resistance within each geographic region. To demonstrate that a standardized methodology could be used to provide cross-comparable molecular surveillance of AMR genes, we initiated a pilot study to test four genera of diarrheal pathogens for > 200 ARDs. Human clinical isolates were obtained from five geographic locations spanning four continents (North and South America, Southeast Asia, and North and East Africa). Not surprisingly, testing in this manner elucidated some generalized trends in AMR in the three Enterobacteriaceae in various geographic regions.

## 2. Results and Discussion

The United States (US) Department of Defense has established its Global Emerging Infections Surveillance and Response System (GEIS) to provide geographically relevant disease surveillance outside the continental US. While the GEIS centralized laboratories have developed standardized protocols for molecular characterization of infectious diseases, many of the surveillance laboratories implement their own protocols for disease detection, identification, and analysis, making comparisons between partner laboratories challenging. For this reason, we sought to demonstrate a harmonized strategy for molecular AMR surveillance in isolates collected at the US Centers for Disease Control and Prevention (CDC) and four GEIS laboratories with their own laboratory-specific and/or narrow spectrum protocols.

In this study, we tested a set of diarrheal isolates collected at clinical sites in the USA, Cambodia, Egypt, Peru, and Kenya. Isolates included *Campylobacter* spp., *Escherichia coli*, *Salmonella*, and *Shigella* spp. These genera were chosen to reflect the top four global bacterial diarrheal pathogens and were responsible for over 430,000 deaths in 2016 [3]. Up to 32 strains from each of the four genera were selected on the basis of phenotypic tetracycline (TET) resistance. Effective against a wide variety of bacteria and protozoan parasites, TETs are first-line antimicrobials used worldwide as therapeutics, are among the most commonly used drugs in agriculture and aquaculture [13,18,27,28], and have been categorized as highly important antimicrobials—with tigecycline considered a critically important antimicrobial [29]. As a result of this widespread selective pressure, ARDs for TET are now the most common of those found within the healthy human fecal microbiome in both industrialized and non-industrialized nations [30]. As TET ARDs are often co-localized on mobilizable elements with other ARDs, we posited that phenotypic TET resistance would serve as an appropriate selection criterion for surveillance of both TET-specific ARDs and those directed against other categories of antimicrobials.

### 2.1. Overall Population Characteristics

**Figure 1:**
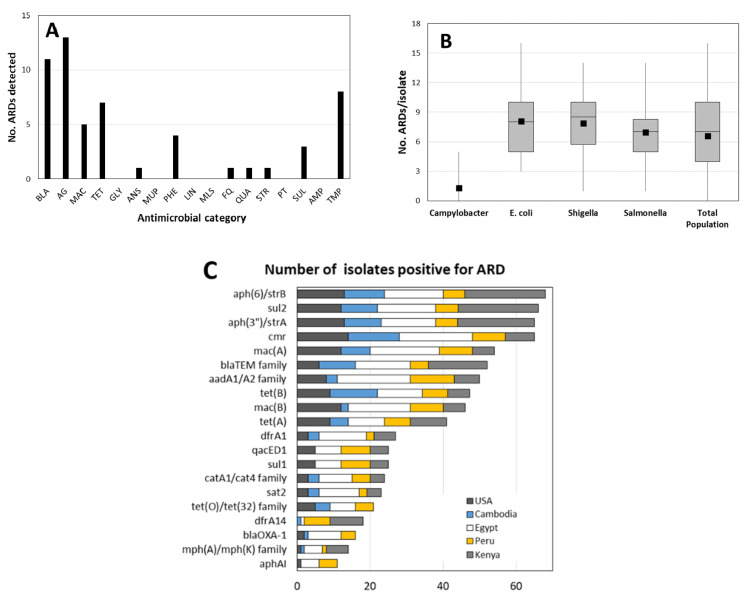
(**A**) Number of antimicrobial resistance determinants (ARDs) detected in tested population, by category; numbers in parentheses indicate total ARDs represented on microarray for that category. BLA = β-lactams (53); AG = aminoglycosides (44); MAC = macrolide (40); TET = tetracyclines (38); GLY = glycopeptides (13); ANS = ansamycins (1); MUP = mupirocin (1); PHE = phenicols (20); LIN = lincosamides (6); MLS = macrolides/lincosamides/streptogramins (13); FQ = fluoroquinolones (4); QUA = quaternary amines (2); STR = streptothricin; PT = platensimycin + platencin (1); SUL = sulfonamides (3); AMP = antimicrobial peptides (1); TMP = diaminopyrimidine. (**B**) Number of ARDs detected per isolate. (**C**) Prevalence of unique ARDs detected in >10 isolates.

We detected a total of 55 different ARDs conferring resistance to eleven different categories of antimicrobials: β-lactams, aminoglycosides, macrolides, TETs, phenicols, streptothricins, sulfonamides, diaminopyrimidine (trimethoprim), ansamycins, and quaternary amines (Figure 1A). None of the thirteen glycopeptide, ten streptogramin, eight macrolide/lincosamide/streptogramin (MLS) resistance determinants, or ten multidrug efflux pumps were detected in the tested population. All isolates tested were negative for the following carbapenemase genes: *bla*_GES_ (including extended-spectrum β-lactamase variants), *bla*_IMI_, *bla*_IMP_, *bla*_KPC_, *bla*_OXA-23_, *bla*_OXA-24_, *bla*_OXA-51_, *bla*_OXA-58_, *bla*_SIM_, *bla*_SME_, *bla*_VIM_. However, genes for OXA-48- and NDM-type carbapenemases were detected in one isolate each (described below). Given the TET-resistant selection criteria for inclusion in the study, we were somewhat surprised that fifteen isolates (two *Campylobacter* spp., three *Shigella* spp., four *E. coli*, and six *Salmonella*) were negative for all 38 TET-specific ARDs represented on the microarray. However, phenotypic TET resistance can arise from multiple intrinsic mechanisms not interrogated or detected by the microarray used here (reviewed by [31,32]): elimination or reduced expression of some outer membrane proteins, constitutive or over-expression of non-specific active efflux or *araC* family activators, and mutations of TET binding sites on rRNA or ribosomal proteins. Furthermore, at least 20 recently documented or predicted TET ARDs are not included in the content of the Antimicrobial Resistance Determinant Microarray (ARDM) v.2 used in this study [33,34,35,36].

The number of ARDs per strain ranged from none (several *Campylobacter* spp. strains) to sixteen, with median and mean values of seven and 6.58 genes per isolate, respectively (Figure 1B). Among the Enterobacteriaceae, 91% harbored genes which were predicted to cause resistance to at least three categories of antimicrobials (Table 1, top).

The genes most commonly observed amongst all of the tested isolates were *strA, strB*, *sul2*, and *cmr*, which were all detected in at least 40% of all isolates and in over half of the Enterobacteriaceae isolates tested (Figure 1C). The relative proportion of isolates harboring each gene varied by both genus and geographic location (Table 1, bottom); geographic differences will be discussed in greater depth in Section 2.3.

### 2.2. AMR Genotypes Detected in Each Genus

#### 2.2.1. *Campylobacter* spp.

*Campylobacter* spp. cause diarrheal disease in over 150 million persons each year globally, resulting in 75,000 deaths [3,37]. Though the true incidence of campylobacteriosis is poorly described, its global prevalence has risen in parallel with increasing resistance to commonly used antimicrobials [37,38]. Of the 23 *Campylobacter* spp. isolates tested, fourteen were *C. jejuni*, six were *C. coli*, and three belonged to other or undefined species.

**Figure 2:**
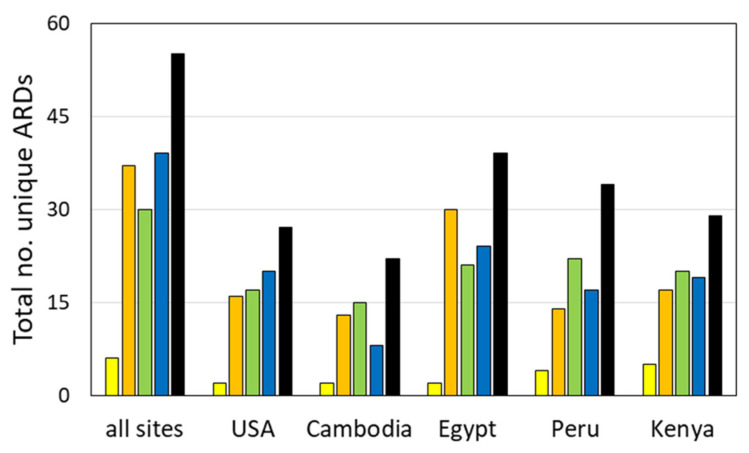
Numbers of unique ARDs observed for *Campylobacter* spp. (yellow), *E. coli* (orange), *Shigella* spp. (green), *Salmonella* (blue), and all species (black) detected in isolates from each collection site.

Overall, *Campylobacter* spp. isolates had both a more limited variety of unique ARDs detected (six in total; Figure 2) and a more limited number of genes detected per isolate than the other three genera (*p* < 0.001) (Table 2, Figure 1B). Resistance in *Campylobacter* is commonly conferred by point mutations, active efflux mechanisms, and the intrinsic low permeability of their cell membranes. Therefore, the number of horizontally transferred ARDs described in *Campylobacter* is limited to a few genes; coverage for *Campylobacter* spp. and other ε-proteobacteria accounts for < 1% of total microarray content [39].

All but three isolates harbored *tet*(O)/*tet*(32) (83%), the most common ARD for TET resistance in *Campylobacter* spp. Two samples were negative for all 38 TET resistance determinants on the microarray, and the third was positive for *tet*(M). It is likely that the two negative samples harbored *tet*(44) [40] (not included in microarray content) or that TET resistance arose from rRNA sequence mutations, constitutive expression of *cmeABC*, or over-expression of one or more efflux pumps [41]. Constitutive expression of *cmeABC* also contributes to fluoroquinolone resistance, which was also observed in 65% of the *Camplyobacter* spp. isolates where phenotypic data were reported. The *tet*(M)-positive sample was potentially a false-positive, as this gene has never been detected in *Campylobacter* spp.; however, this gene has the broadest host range of all TET ARDs [27,42]. The *tet*(M) reference sequence is 80% identical to *tet*(O)/*tet*(32) over the entire gene and some regions have significantly higher identity. Understanding that the microarray used here requires approx. 85–90% sequence identity for detection of similar genes (or families of genes) [43,44], it is unclear whether the redesign of probes for this gene will improve microarray specificity. 

Four additional ARDs were detected amongst the *Campylobacter* spp. isolates. Two isolates co-harbored *aadE*, *aphA3* family, and *sat4*, which are often found as part of resistance clusters in *C. coli* and *C. jejuni* plasmids and in the chromosome of some *C. jejuni* strains [45,46]. *Erm*(B) was also observed in one of these isolates; this gene confers high-level resistance to macrolides such as azithromycin (AZM), which is a first-line therapeutic for campylobacteriosis in locations where fluoroquinolone (FQ) resistance is prevalent [9,10]. Since *erm*(B)-*aadE*-*aphA3*-*tet*(O) clusters have been documented in a number of plasmids [47], co-carriage of these four genes suggests that this strain may harbor a plasmid. The prevalence of *erm*(B) amongst *Campylobacter* spp., while not historically high, has recently increased in East Asia and Southern Africa [47,48].

Other ARDs occasionally observed in *Campylobacter* spp. were either not detected (*tet*(S), *aph(2)-Ic*) or not included in the microarray content (*tet*(44), *ant(6)-Ib*, *bla*_OXA-61_; *aacA*/*aphD*, *aac*, *aad6*, *aad9*, *aph(2)-3a*).

#### 2.2.2. *E. coli*

Diarrheagenic *E. coli* are responsible for over 100 million illnesses and 60,000 deaths each year [49]. Of the 30 *E. coli* samples provided, 12 were categorized into pathotypes based on their mechanism of virulence: enterotoxigenic—three from Kenya, two from Cambodia, two from Peru; enteroaggregative—one each from Kenya and Cambodia; enterohemorrhagic—one from Kenya; enteropathogenic—one each from Cambodia and Peru. Pathotypes for the remaining samples were not provided.

Thirty-seven unique ARDs were detected among the tested *E. coli* strains (Table 3, Figure 2). As expected, TET ARDs were harbored in > 80% of the *E. coli* strains: *tet*(A) (nine isolates, 30%), *tet*(B) (16 isolates, 53%), *tet*(C) (two isolates, 6%), and *tet*(D) (one isolate, 3%). Two Egyptian isolates harbored both *tet*(A) and *tet*(B) genes, and four isolates were negative for all 38 TET genes represented on the microarray. Other genes with high prevalence included bla_TEM_ (64%), *strA* and *strB* (70% each), *mac*(A) and *mac*(B) (77% and 67%, respectively), *cmr* (83%), and *sul2* (67%) (Figure 1C). Of potential clinical concern was the presence of *mph*(A)/*mph*(K) in eight isolates (27%); genes in this family confer resistance to AZM, an alternative therapeutic for severe travelers’ diarrhea and shigellosis in both children and adults [9,10,50,51,52]. While AZM is relatively ineffective for *E. coli* infections, interspecies transfer of AZM resistance plasmids has been documented between *E. coli* and *Shigella* spp. [53,54,55], further supporting the role of *E. coli* as a potential reservoir for ARDs causing clinically relevant drug resistance in *Shigella* spp.

Of additional concern, six of the *E. coli* strains (20%) harbored a gene encoding a CTX-M type of extended-spectrum β-lactamase (ESBL). Furthermore, some *bla*_TEM_ and *bla*_SHV_ alleles—detected in 63% of all *E. coli* strains—may also encode ESBLs. ESBL phenotype is conferred to TEM and SHV β-lactamases by a wide variety of single nucleotide polymorphisms (SNPs; see https://externalwebapps.lahey.org/studies/webt.aspx for classification and nomenclature of *bla*_TEM_ and *bla*_SHV_ alleles). The microarray used here is unable to detect these SNPs and therefore cannot identify the *bla*_TEM_ and *bla*_SHV_ alleles. We anticipate that our estimate of approx. 20% ESBL carriage in *E. coli* (i.e., from *bla*_CTX-M_-positive isolates) may be an underestimate; analogous situations hold for *Salmonella* and *Shigella* spp. strains. These results illustrate that techniques offering greater sequence resolution, such as amplicon or genome sequencing, may provide additional information that is important for molecular surveillance.

The *bla*_NDM_ carbapenemase gene was detected in a single Egyptian *E. coli* isolate. Coincidentally, many of the ARDs often found on plasmids with *bla*_NDM_ (e.g., *bla*_OXA-1_ family, *bla*_TEM_ family, *bla*_CTX-M-1_ family, *aadA1/A2*, *qnrS*, *aac(6’)-Ib*) were also observed in this isolate, suggesting that this gene might be plasmid-borne. However, we did not attempt to isolate or sequence plasmids from any of the isolates tested.

Four *E. coli* strains harbored both *qacEΔ* and *sul1*, which are components of the 3′-conserved region of many, but not all, class 1 integrons. Similarly, three *E. coli* isolates co-carried three genes commonly associated with class 2 integrons—*dfrA1*, *aadA1/A2*, and *sat2*—suggesting the presence of class 2 integrons within these isolates. Integrase-specific PCRs confirmed the presence of class 1 (*intI1*) and class 2 (*intI2*) integrons within all samples that co-carried these ARDs (13% and 7%, respectively). These numbers likely underestimate the carriage of class 1 and class 2 integrons within this population, as they did not account for integrons with alternative structures lacking one or more “marker” genes [39,56,57,58]. Furthermore, some samples deemed microarray-positive for these “marker” genes did not have sufficient volume for the confirmatory PCRs.

Other possible gene assemblages were detected. Positive results for *strA*, *strB*, and *sul2* were highly correlated (*p* < 0.001) and co-carriage was observed in 64% of the *E. coli* strains. These genes are commonly observed as a cluster in plasmids of clinical Enterobacteriaceae isolates, as well as in integrative and conjugative elements in *Vibrio* spp. [59,60]. Similarly, *mac*(A) was highly correlated with *mac*(B) (*p* < 0.001)—not surprising, since these genes are typically found in a single operon [61]. Carriage of one of both of these genes was also correlated with the presence of *cmr* (*p* < 0.001); while also part of the *E. coli* chromosome, *cmr* is found up to 40 kb away from the *macAB* operon.

#### 2.2.3. *Shigella* spp.

*Shigella* spp. have a low infectious dose (only 10–100 organisms) compared to other diarrheagenic organisms and were the second leading cause of global diarrheal mortality in 2016 [3,62]. In contrast to most illnesses arising from other gastrointestinal pathogens, antimicrobials are recommended to reduce the clinical course of shigellosis and to prevent transmission. The increasing prevalence and spread of *Shigella* spp. resistant to first-line therapeutics are therefore of tremendous concern, particularly in Asia and Africa [62].

The 45 shigellae tested here were roughly divided between *S. flexneri* (13 isolates), *S. sonnei* (13 isolates), and *Shigella* sp. (12 isolates), with the remaining strains split between *S. boydii* (four isolates) and *S. dysenteriae* (three isolates). Thirty unique ARDs were detected amongst the *Shigella* spp. isolates (Table 4)—somewhat more limited than in *E. coli* or *Salmonella* (Figure 2). The per-isolate carriage rate was higher in *Shigella* spp. (Figure 1B, *p* < 0.025).

As shigellae and *E. coli* are very closely related [63], we were not surprised to see many of the same ARDs detected in *Shigella* spp. with similar frequencies as in *E. coli*: *strA* (56%), *strB* (58%), *mac*(A) (64%), *mac*(B) (58%), *cmr* (82%), and *sul2* (64%) (Figure 1C). TET ARDs detected amongst the shigellae were limited to *tet*(A) (39%) and *tet*(B) (59%); three strains were negative for all tested TET ARDs. Plasmid-borne FQ ARD, *qnrS*, was observed in two isolates, and the ansamycin resistance gene, *arr*, was detected in a Cambodian *S. dysenteriae* isolate. Notably, *bla*_CTX-M-1_ and *bla*_CTX-M-9_ family genes were detected at a much lower rate in the *Shigella* spp. strains than in *E. coli*—2% and 5%, respectively. Other notable differences between *Shigella* spp. and the closely related *E. coli* were the lower carriage of *bla*_TEM_ (27% versus 63%) and *mph*(A)/*mph*(K) (9% versus 27%) and higher carriage of the *bla*_OXA-1_ family (34% versus 3%) in *Shigella* spp. isolates.

As with *E. coli*, the presence of some sets of genes was highly correlated: *strA*, *strB*, and *sul2* (*p* < 0.001); *mac*(A), *mac*(B), and *cmr* (*p* < 0.001); *qacEΔ* and *sul2* (*p* < 0.001), and *aadA1/A2*, *sat2*, and *dfrA1* (*p* < 0.005). *IntI1*-specific PCR confirmed the presence of class 1 integrons in 7% of the *Shigella* spp. strains, similar to observations in *E. coli*. However, *intI2*-specific PCR detected class 2 integrons in a much larger proportion of shigellae (29%). As only those isolates with integron-associated markers were tested, these numbers are likely underestimates. 

Twelve *Shigella* spp. strains (27%) carried the same four genes found in the *Shigella* resistance island (SRL; *aadA1*, *bla*_OXA-1_, *tet*(B), and *catA1*), a chromosomal pathogenicity island found in *S. flexneri*, *S. sonnei*, and *S. dysenteriae* with wide global distribution [64]. No samples from the other three genera carried this unique combination of genes.

#### 2.2.4. *Salmonella* spp.

*Salmonella* gastroenteritis affects nearly 200 million persons, causing over 80,000 deaths in 2016 [62]. As typhoid fever can also cause diarrhea in young children and adults with HIV [65,66], four *S. enterica* subsp. enterica serotype Typhi isolates were included amongst the 32 samples tested here. Serotypes for 21 additional non-typhoidal *Salmonella* (NTS) isolates were also provided: five isolates of serotype B (four from Cambodia, one from USA) and one strain each of serotypes Newport, Anatum, Heidelberg, Dublin, and Agona (all USA isolates); one isolate each of serotype A and O (Kenya); one isolate of serogroup 3 (Egypt), and three isolates each from serogroups B and D, and two from serogroup C1 (Peru).

Thirty-eight ARDs were detected among the *Salmonella* strains (Figure 2, Table 5). Eighty-five percent of the *Salmonella* isolates harbored a TET ARD, comprising a wider variety of TET resistance genes than observed in *E. coli* or *Shigella* spp.: *tet*(A) (47%), *tet*(B) (22%), *tet*(C) (3%), *tet*(D) (6%), and *tet*(G) (6%). The following genes were also detected in a significant proportion of the *Salmonella* isolates: *strB* (63%), *bla*_TEM_ (56%), *strA* (56%), *qacEΔ*, (53%), *sul1* (53%), *sul2* (50%), and *aadA1/A2* (50%) (Figure 1C).

A variety of genes not detected amongst the other Enterobacteriaceae isolates was observed in a few *Salmonella* strains (< 10%): *bla*_CMY/LAT_, *bla_PSE/CARB_*, *bla*_OXA-48_-like, *aac(3)-Id*, *aac(3)-III*, *aadA7*, *rmtD*, *tet*(G), *floR*, and *sul3*.

Of clinical concern, genes encoding CTX-M-type ESBLs were detected in 12% of the *Salmonella* isolates, conferring resistance to first-line therapeutics for NTS diarrhea in areas with multidrug resistance (MDR) [9]. On the other hand, *rmtD*—detected in two isolates—is not clinically relevant for *Salmonella*-derived infections but confers resistance to all aminoglycosides and can be transferred horizontally to other species for which aminoglycosides serve as common therapeutics. Also alarming was detection of the carbapenemase gene, *bla*_OXA-48_, in a Kenyan *Salmonella* isolate. While its presence in Enterobacteriaceae in East Africa has recently been reported [67,68,69,70], to our knowledge, this is the first description of an OXA-48-like-containing diarrheal isolate in Kenya.

The *qacEΔ* and *sul1* genes were co-harbored in over half of the *Salmonella* strains, and the presence of *intI1* in these strains was confirmed by PCR. The prevalence of class 1 integrons in the *Salmonella* strains was therefore significantly higher (51%) than observed in *E. coli* (13%) or *Shigella* spp. strains (7%). However, no *Salmonella* isolates co-harbored the combination of *aadA1/A2* + *sat2* + *dfrA1*, supporting previous observations that class 2 integrons are infrequently harbored by *Salmonella* [71,72,73].

Several isolates harbored ARDs commonly associated with *Salmonella* genomic island 1 (SGI1; *aadA1/A2*, *qacEΔ*, *sul1*, *floR*, *tet*(G), *bla*_PSE/CARB_) [74] or its SGI1-K variant (*aac(3)-id*, *aadA7*, *qacEΔ*, *sul1*, *tet*(A), *bla*_TEM_) [75].

### 2.3. Geographic Trends

**Figure 3:**
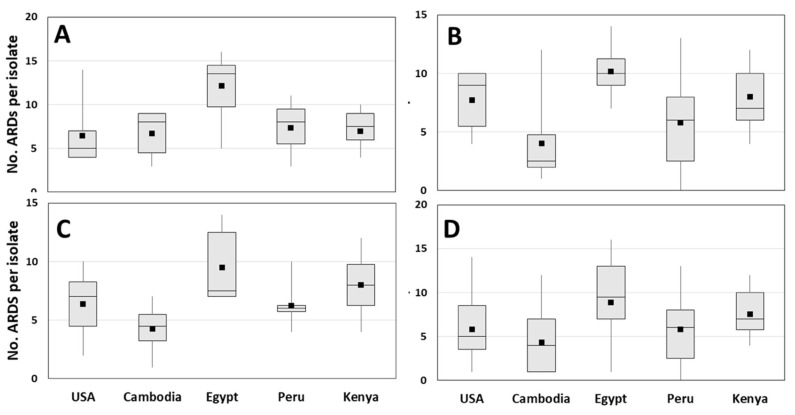
Box and whisker charts showing number of unique ARDs per isolate for (**A**) *E. coli*; (**B**) *Shigella* spp.; (**C**) *Salmonella*; (**D**) each population as a whole (including *Campylobacter* spp.). Black squares in each chart indicate mean values.

**Figure 4:**
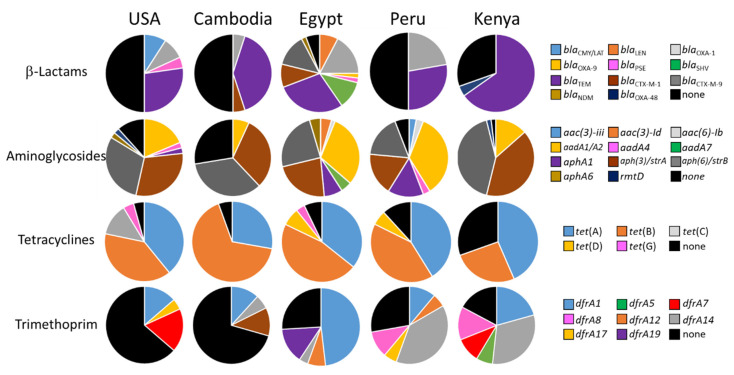
Pie charts showing carriage rates of ARDs conferring resistance to β-lactams, aminoglycosides, tetracyclines, and trimethoprim among *E. coli*, *Shigella* spp., and *Salmonella*. Black pie slices indicate the percentage of isolates that were negative for all tested ARDs in that category. Note that many strains carried multiple β-lactamase and aminoglycoside ARDs.

As a pilot-scale study, the numbers of isolates obtained per genus and per site hindered attempts to draw strong statistical conclusions from the data. However, a number of general trends were observed. Of the five collection sites surveyed, Egypt had the largest total number of ARDs detected in *E. coli* (30), in *Salmonella* (23), and overall (39) (Figure 2, Table 2, Table 3, Table 4, Table 5 and Table 6). Not surprisingly, the number of unique ARDs on a per-isolate basis was also highest in Egyptian samples for the three Enterobacteriaceae, suggesting that Egyptian isolates were more likely to possess MDR (Table 1 and Table 6, Figure 3). The high ARD carriage rate in the Egyptian isolates was largely due to the high diversity and rates of carriage of genes encoding β-lactamases (90%) and aminoglycoside modifying enzymes (100%) (Table 2, Table 3, Table 4, Table 5 and Table 6, Figure 4). Over half harbored at least three aminoglycoside ARDs and nearly half of those with β-lactamases encoded a CTX-M-type ESBL. In comparison, 30% to 50% of Enterobacteriaceae isolates from the USA, Cambodia, Peru, or Kenya were negative for all β-lactamase genes, and fewer aminoglycoside ARDs were present in each. The high prevalence of *bla*_CTX-M-1_ and *bla*_CTX-M-9_ families in Egyptian isolates also supports previous observations of high ESBL carriage [76,77] and may be related to the preferential consumption of extended-spectrum cephalosporins over narrow-spectrum penicillins in Egypt [78,79]. Furthermore, high carbapenem consumption in Egypt [79] may also have provided selective pressure for the single *bla*_NDM_-positive sample, also observed within this population. Of additional note, a significant percentage (27%) of the Egyptian Enterobacteriaceae harbored genes conferring resistance to AZM (two isolates with *ere*(A), five with *mph*(A)/*mph*(K)); Kenya was the only other site where AZM ARDs were carried at a similar rate (30% in Enterobacteriaceae).

Cambodia had the lowest total number of unique ARDs detected for the population as a whole and for each of the four genera tested. The lowest number of ARDs per isolate for *Shigella* spp., *Salmonella* spp., and all genera combined were also observed in the Cambodian samples (Figure 3). Interestingly, neither the high levels of phenotypic resistance nor genotypic profiles observed in two previous studies of fecal isolates from Southeast Asia [80,81] were evident from the current study. This apparent contradiction suggests that the current study may have underestimated the true prevalence of AMR genes in the Cambodian samples. One explanation for this possible underestimation is that the Cambodian samples were isolated, extracted, prepared for microarray analysis, and subsequently tested on the ARDM v.2 microarray in Naval Medical Research Unit-2 (NAMRU-2) facilities. DNA from isolates collected at the other sites (USA, Egypt, Kenya, and Peru) was extracted at the site of collection and then the DNA was shipped to the US Naval Research Laboratory (NRL) in Washington, DC, USA; these extracted DNA preparations were then further prepared and analyzed on the microarrays within NRL facilities. Slight differences in instrumentation or handling between NAMRU-2 and NRL (e.g., ramp speed during fragmentation steps, cold blocks versus ice baths) may have affected the size and distribution of the tested nucleic acids, resulting in altered test sensitivity [82]. It should be noted, however, that a study of Cambodian wound isolates performed at the same time documented higher rates of carriage of specific ARDs in *E. coli* than observed here [83].

Other interesting observations included the detection of ARDs associated with SGI1-K (*Salmonella*: *aac(3)-id*, *aadA7*, *qacEΔ*, *sul1*, *tet*(A), *bla*_TEM_; [75]) or plasmid pSLBT (*E. coli*: *aadA1*, *strA*, *strB*, *bla*_TEM_, *catA1*, *dfrA1*, *sul1*, *sul2*, *qacEΔ*; [84]) in African isolates only. Both assemblages, SGI1-K and pSLBT, were originally described in Nigeria and Kenya, respectively [75,84]. While we did not attempt to confirm the presence of either SGI1-K or pSLBT (or pSLBT-like plasmids) in any of these strains, the African origin of these strains suggests that mobilizable elements such as these may be circulating within East and North Africa.

Twenty-seven percent (27%) of the *Shigella* spp. isolates also harbored ARDs associated with SRL, but carriage rates were not consistent between collection sites: the rates of co-carriage were much higher amongst Egyptian and Peruvian strains (58% and 50%, respectively, versus 0–14% for the other sites). These different carriage rates may be related to the prevalence of the various global *Shigella* spp. lineages (and related absence/presence of SRL) within each site [85].

A correlation between national antimicrobial consumption and prevalence of ARDs at specific sites was sometimes, but not always, observed. For example, the high prevalence and wide variety of *sul* and *dfrA* genes in Kenyan Enterobacteriaceae (Table 2, Table 3, Table 4 and Table 5, Figure 4) may potentially be due to selective pressure from the widespread prophylactic use of SXT for HIV-infected individuals [86]. The high prevalence of ESBLs and the observation of *bla*_NDM_ amongst Egyptian samples, where these antimicrobials are widely consumed, serve as a second example. On the other hand, we detected *bla*_OXA-48_ in an isolate from Kenya, where carbapenems are expensive and presumably not widely used [79,87]. These observations support other studies reporting significant increases in carbapenemase-producing Enterobacteriaceae in East Africa, in spite of this presumably low selective pressure [69,88]. While a large number of plasmids carrying *bla*_OXA-48_ and related carbapenemase genes have few or no additional ARDs, many of these plasmids have minimal associated fitness cost and can be stably maintained in the absence of selective pressure [89,90,91]. Others have documented the maintenance of plasmids harboring larger numbers of ARDs in the absence of selective pressure [92,93,94].

A key limitation of the current study is the low numbers and variability of isolates of each species obtained from the five sampling sites, constraining our ability to perform robust statistical comparisons. This limitation is further compounded when pathotypes (*E. coli*), species (*Shigella*, *Campylobacter*), serotypes (*Salmonella*), or sampled populations (e.g., hospital inpatients versus outpatients) vary between sites or were not fully characterized. Furthermore, Cambodian and Kenyan subject populations (mean subject ages 7.9 and 8.0 years, respectively) tended to be younger than those from the USA or Peru (mean subject ages 30.9 and 22.4 years, respectively; see Supplementary Materials). Another key concern was the difference in methods used to extract DNA, as partnering labs were asked to provide DNA extracted as per their standard operating procedures, which we erroneously assumed would be uniform between sites; future studies should standardize the extraction procedures as well as the analysis. Though rapid and easy, the boil method (Peru) may not provide DNA of the same quality as commercial kits. Furthermore, the kit used to prepare Kenyan samples is designed to enrich for plasmid and cosmid DNA and may have affected the recovery of chromosomal DNA; for example, some chromosomal ARDs (*mac*(A), *mac*(B), *cmr*) were detected at lower-than-expected rates in Kenyan samples. More importantly, with regard to study design, it is also unclear whether the sample set used here—selected for TET resistance—accurately reflects the overall prevalence of various ARDs within the larger populations, including both resistant and susceptible pathogens from these collection sites; TET ARDs are commonly found in assemblages with other ARDs. Future study designs will emphasize a single species or serotype, with a larger number of samples collected from each site. Integrating a whole genome sequencing approach into a follow-on study would improve both the analytical depth and breadth. Whole genome sequencing—while more computationally intense—would enable rapid discrimination of closely related alleles or variants conferring different phenotypes (e.g., *aac(6’)-ib* versus *aac(6’)-ib-cr*) and detect chromosomal mutations conferring resistance (e.g., FQ resistance from *gyrA* or *parC* mutations, TET resistance from rRNA gene mutations, etc.); the microarray used here does not provide this capability. Next generation sequencing can further provide high resolution epidemiological tracking and “One Health” linkages [95,96], provide context for genes, and even detect promoter modifications that significantly affect gene expression [97,98]. However, a key limitation of any molecular technology approach is that genotype may not always be predictive of phenotype if differential expression and/or functionality of expressed proteins is not known or fully characterized. Furthermore, cooperative interactions amongst members of an ecosystem consortium can significantly affect individual species’ phenotypic traits and survivability in native environments [22,99,100,101,102]. Therefore, while molecular techniques are useful for AMR surveillance, they should be complemented with approaches that address additional mechanisms for AMR and survival and (potentially uncharacterized) contributions of components of the ecosystem consortium.

The current study does address the presence of a large number of resistance markers covering multiple classes of antimicrobials across a diverse collection of organisms comprising four genera using a standardized and broad-spectrum detection platform. This approach allowed us to detect possible assemblages and make some surprising observations. With the exception of *Campylobacter* spp., a high rate of ARD carriage in all populations was observed (average = 6.58 genes/isolate; median = 7 genes/isolate among the Enterobacteriaceae). While the prevalence of ESBLs was alarming—especially in Egypt—we observed only two samples that harbored carbapenemase genes; other studies have reported much higher levels of carbapenemase-producing Enterobacteriaceae within fecal samples [103,104].

Genes conferring resistance to AZM were observed in > 10% of the entire population, with Enterobacteriaceae strains from Egypt and Kenya having the highest rates of carriage (27% and 30%, respectively). The high prevalence of these ARDs is particularly concerning, as AZM has replaced FQs as a first-line therapeutic for moderate travelers’ diarrhea in Southeast Asia and for severe travelers’ diarrhea globally [105]. The low prevalence of the FQ resistance gene, *qnrS*, observed from all collection sites mirrored results from other studies [106,107,108]. This and other plasmid-mediated quinolone resistance (PMQR) genes reduce susceptibility to quinolones, albeit not always to the level of clinical resistance [98,109]. High-level resistance to first-line FQ therapeutics is generally conferred by point mutations in *gyrA* and *parC* in Enterobacteriaceae, which are not detectable by the microarray technology used here; the ability to interrogate samples for such point mutations would be an advantage of any sequencing-based approach.

While the selection criteria used here (TET resistance) may have artificially raised the overall prevalence of ARDs directed against other antimicrobials, we were somewhat surprised to see such a broad variety and high carriage rate of many ARDs in isolates collected from all sites. In particular, the large numbers of strains among the Egyptian samples positive for ARDs directed against WHO’s critically important antimicrobials (carbapenems, third generation cephalosporins, AZM) suggest an urgent need for improving awareness of AMR, more in-depth surveillance for resistant pathogens, and improved antimicrobial stewardship within this region.

## 3. Materials and Methods

### 3.1. Isolates

A total of 23 *Campylobacter* spp., 30 *Escherichia coli*, 32 *Salmonella* spp., and 45 *Shigella* spp. diarrheal isolates were selected from pre-existing collections at the US Centers for Disease Control and Prevention (CDC, Atlanta, GA, USA; *n* = 27), US Army Medical Research Directorate, Africa/Kenya Microbiology Hub, Kericho (USAMRD-A/K, Kericho, Kenya; *n* = 24), US Naval Medical Research Unit No. 2 (NAMRU-2, Phnom Penh, Cambodia; *n* = 25), US Naval Medical Research Unit No. 3 (NAMRU-3, Cairo, Egypt; *n* = 32), and US Naval Medical Research Unit No. 6 (NAMRU-6, Lima and Cusco, Peru, *n* = 24, NAMRU6.2017.0012 protocol). Collection dates ranged from 2006 to 2013 (Supplementary Materials), and isolates were identified as previously described [110]; prior to use in this study, all isolates were stripped of all identifiers that could be used to trace them back to an individual. Isolates were selected based on tetracycline (TET) non-susceptibility, using breakpoints determined by Clinical and Laboratory Standards Institute (CLSI) standards [111]. Limited phenotypic antimicrobial susceptibility profiles and isolate metadata were available for some, but not all, isolates (Supplementary Materials). We therefore had an insufficient number of isolates to allow statistically robust genotype–phenotype correlations.

### 3.2. Processing and DNA Hybridization

Genomic DNA was extracted from archived stool isolates at each collection site using the following: the boil method (used in Peru [112]), Masterpure DNA and RNA Complete Purification Kit (Epicentre Biotechnologies, Madison, WI, USA; used in Egypt), R.E.A.L. Prep Kit (used in Kenya), DNeasy Blood and Tissue Kit (used in USA), or QIAamp DNA mini (used in Cambodia) (last three kits from QIAGEN, Germantown, MD, USA; USA), according to manufacturers’ instructions.

With the exception of Cambodia, all subsequent steps were performed in NRL facilities (amplification, labeling, hybridization, microarray interrogation) as previously described [113]; all steps for preparation and analysis of Cambodian samples were performed on-site in NAMRU-2 facilities (Phnom Penh, Cambodia). Each sample was amplified using 10 ng of template DNA and Illustra GenomiPhi v.2 DNA Amplification Kits (GE Healthcare, Pittsburgh, PA, USA), as per the manufacturer’s instructions. After quantification using Qubit 2.0 fluorometer (Thermofisher, Rockland, IL, USA), 2 µg of the whole genome amplicons were fragmented for 1 min at 37 °C with 2.7 units DNase I (total volume of 60 µL; enzyme and buffer from GeneChip Resequencing Assay Kit, AffyMetrix, Santa Clara, CA, USA), incubated for 10 min at 95 °C to inactivate the DNase I, then purified on DNA Clean & Concentrator-5 columns (Zymo Research, Irvine, CA, USA). Fragmented, purified DNA was then biotinylated using ULS Platinum Bright Biotin Nucleic Acid Labeling Kits (Kreatech Diagnostics, Durham, NC, USA; 10 µL reaction volume), as per the manufacturer’s instructions. The resulting biotinylated fragments were then applied to pre-hybridized ARDM v.2 microarrays (Customarray, Bothell, WA, USA [39]), hybridized overnight at 60 °C, and labeled with 1000 × diluted multimeric streptavidin-horseradish peroxidase (65R-S104PHRP; Fitzgerald Industries, North Acton, MA, USA), washed, processed, and electrochemically interrogated using the ElectraSense reader (Customarray), as previously described [39]. The content of the ARDM v.2 microarray includes 25- to 35-mer probes directed against 238 gene sequences (between 8 and 10 probes per gene) predicted to confer resistance to 15 categories of antimicrobials: β-lactams (*n* = 46 genes), aminoglycosides (*n* = 42), macrolides (*n* = 27), lincosamides (*n* = 22), streptogramins (*n* = 18), quaternary amines (*n* = 2), ansamycins (*n* = 1), diaminopyrimidines (*n* = 28), antimicrobial peptides (*n* = 1), tetracyclines (*n* = 38), phenicols (*n* = 10), glycopeptides (*n* = 12), platensimycin/platensin (*n* = 1), fluoroquinolones (*n* = 4), sulfonamides (*n* = 3). Many of the macrolide, lincosamide, and streptogramin ARDs overlap in specificity. Samples with > 85–90% gene sequence identity to the reference sequence can successfully hybridize to the microarray, allowing a broader variety of ARDs—i.e., families of genes—to be detected. However, the microarray is unable to distinguish between these similar genes. A gene was deemed present if at least 50% of its representative probes had signals above the 95% probe threshold (mean signal from lowest 2128 probes + 3 SD) or if ≥ 70% of its probes had signals above either of two less stringent thresholds (mean signal from lowest 2016 probes + 3 SD or mean signal from lowest 2128 probes + 2 SD) [39,113]. Using these algorithms, the sensitivity and specificity of the microarray were calculated as 96.3–100% and 97.9–100%, respectively [39,44].

### 3.3. Confirmatory PCR

Where sufficient sample was available, PCR amplifications confirming the presence of *intI1* and *intI2* [114] were performed on all isolates that were positive for *qacEΔ*1 + *sul1* or *aadA1/A2* + *sat2* + *dfrA1*, respectively. Retrospective analysis of samples from Cambodia could not be performed.

### 3.4. Statistics

Statistical comparisons between populations were performed using two-tailed *t*-tests and analysis of variance (ANOVA) when normally distributed and using Mann Whitney rank sum test when not normally distributed. Chi-square tests were used to compare binomial proportions (presence/absence) in independent samples (2 × *n* contingency tables). *P* < 0.05 indicates significance.

## 4. Conclusions

This pilot study—using DNA preparations from 130 human diarrheal isolates—demonstrated that a standardized methodology could be used to provide molecular surveillance for over 200 AMR genes. We detected a wide variety of ARDs amongst the tested isolates and observed some generalized trends, although the small sample size limited our ability to make many conclusions with sufficient statistical power. Overall, we detected the greatest numbers and diversity of ARDs in isolates from Egypt and the lowest numbers and diversity in isolates from Cambodia. Though we could not make any conclusions regarding the phenotypes based on the ARDs detected, this preliminary study serves as a valuable starting point for more detailed follow-on investigations. The present study’s findings suggest that the implementation of molecular detection platforms with sufficient breadth—when used in conjunction with complementary techniques—have the ability to improve surveillance efforts that seek to monitor the migration and evolution of MDR organisms over time and space.

## Figures and Tables

**Table 1:** Percentage of isolates with potential resistance to multiple classes of antimicrobial compounds based on their microarray profiles.

**By Genus**								
**No. of Antimicrobial Classes**	***Campylobacter***		***E. coli***		***Salmonella***		***Shigella***	
0	9		0		0		0	
1	74		0		3		4	
2	9		3		6		7	
3	4		17		13		11	
4	4		13		13		4	
5	0		10		25		20	
6	0		13		31		4	
7	0		27		9		31	
8	0		17		0		16	
9	0		0		0		2	
% potentially resistant to:								
≥3 classes	8		97		91		89	
≥6 classes	0		57		40		53	
No. of isolates tested	23		30		32		45	
**By geographic location**								
**No. of Antimicrobial Classes**	**USA**	**Cambodia**		**Egypt**		**Peru**		**Kenya**
0	0	0		0		8		0
1	15	30		16		17		0
2	7	13		6		4		0
3	19	17		0		8		17
4	15	13		3		4		4
5	15	4		9		25		33
6	7	4		16		13		17
7	19	17		22		13		25
8	4	0		28		4		4
9	0	0		0		4		0
% potentially resistant to:								
≥3 classes	78	57		78		71		100
≥6 classes	30	21		66		34		46
No. of isolates tested	27	23		32		24		24

**Table 2:** ARDs detected in *Campylobacter* spp. by geographic location (percent of isolates positive).

ARD	USA (*n* = 5)	Cambodia (*n* = 4)	Egypt (*n* = 6)	Peru (*n* = 7)	Kenya (*n* = 1)
*aadE*	nd *	nd	nd	14%	100%
*aphA3*	20%	nd	nd	14%	100%
*erm*(B)	nd	nd	nd	nd	100%
*tet*(M)	nd	nd	nd	nd	100%
*tet*(O)/*tet*(32)	100%	100%	100%	71%	nd
*sat4*	nd	nd	17%	14%	100%

* not detected.

**Table 3:** ARDs detected in *E. coli*, by geographic location (percent of isolates positive).

ARD	USA (*n* = 7)	Cambodia (*n* = 4)	Egypt (*n* = 8)	Peru (*n* = 3)	Kenya (*n* = 8)
*bla*_LEN_	nd *	nd	13%	nd	nd
*bla*_OXA-1_ family	nd	nd	13%	nd	nd
*bla*_OXA-9_ family	nd	nd	13%	nd	nd
*bla*_SHV_ family	nd	nd	25%	nd	nd
*bla*_TEM_ family	14%	100%	88%	67%	63%
*bla*_CTX-M-1_ family	nd	nd	38%	nd	nd
*bla*_CTX-M-9_ family	nd	nd	38%	nd	nd
*bla*_NDM_	nd	nd	13%	nd	nd
*aac(6)-Ib*	nd	nd	13%	nd	nd
*aadA1/A2*	nd	nd	63%	67%	nd
*aphA1*	nd	nd	25%	nd	nd
*aphA4*	14%	nd	nd	nd	nd
*strA*	57%	75%	75%	33%	88%
*strB*	57%	75%	75%	33%	88%
*aphA6*	nd	nd	13%	nd	nd
*mac*(A)	86%	100%	88%	100%	38%
*mac*(B)	86%	25%	88%	100%	38%
*mph*(A)*/mph*(K)	14%	25%	50%	nd	25%
*tet*(A)	29%	25%	50%	nd	25%
*tet*(B)	43%	75%	50%	33%	50%
*tet*(C)	29%	nd	nd	nd	nd
*tet*(D)	nd	nd	3%	nd	nd
*catA1/cat4*	nd	25%	25%	33%	25%
*cmr*	100%	100%	100%	100%	38%
*qnrS*	nd	25%	13%	nd	nd
*qacE* *Δ* *1*	14%	nd	13%	33%	13%
*sat2*	nd	nd	38%	33%	nd
*sul1*	14%	nd	25%	33%	13%
*sul2*	57%	75%	63%	33%	88%
*dfrA1*	nd	nd	50%	nd	nd
*dfrA5*	nd	nd	nd	nd	13%
*dfrA7*	14%	nd	nd	nd	13%
*dfrA8*	nd	50%	nd	33%	25%
*dfrA12*	nd	nd	13%	nd	nd
*dfrA14*	nd	nd	nd	nd	63%
*dfrA17*	14%	nd	nd	nd	nd
*dfrA19*	nd	nd	25%	nd	nd

* not detected.

**Table 4:** ARDs detected in *Shigella* spp., by geographic location (percent of isolates positive).

ARD	USA (*n* = 7)	Cambodia (*n* = 11)	Egypt (*n* = 12)	Peru (*n* = 6)	Kenya (*n* = 9)
*bla*_LEN_	nd *	nd	8%	nd	nd
*bla*_OXA-1_ family	29%	9%	67%	67%	nd
*bla*_SHV_ family	nd	nd	8%	nd	nd
*bla*_TEM_ family	14%	18%	25%	17%	56%
*bla*_CTX-M-1_ family	nd	nd	8%	nd	nd
*bla*_CTX-M-9_ family	nd	nd	17%	nd	nd
*aac(6)-Ib*	nd	nd	nd	17%	nd
*aadA1/A2*	57%	18%	100%	67%	44%
*aadA4*	nd	nd	nd	17%	nd
*strA*	57%	27%	50%	50%	100%
*strB*	57%	36%	50%	50%	100%
*mac*(A)	86%	18%	100%	100%	33%
*mac*(B)	86%	9%	83%	100%	33%
*mph*(A)*/mph*(K)	nd	nd	nd	17%	33%
*tet*(A)	57%	18%	33%	nd	78%
*tet*(B)	29%	73%	67%	100%	11%
*arr*	nd	nd	nd	17%	nd
*catA1/cat4*	14%	18%	58%	67%	nd
*cmr*	100%	73%	100%	100%	44%
*qnrS*	nd	nd	8%	nd	11%
*qacE*Δ*1*	14%	nd	8%	17%	11%
*sat2*	43%	18%	67%	17%	44%
*sul1*	14%	nd	nd	14%	11%
*sul2*	57%	27%	75%	67%	100%
*dfrA1*	43%	18%	75%	17%	44%
*dfrA5*	nd	nd	nd	nd	11%
*dfrA7*	14%	nd	nd	nd	11%
*dfrA8*	nd	nd	nd	14%	11%
*dfrA14*	nd	9%	8%	33%	11%
*dfrA17*	nd	nd	nd	17%	nd

* not detected.

**Table 5:** ARDs detected in *Salmonella* spp., by geographic location (percent of isolates positive).

ARD	USA (*n* = 8)	Cambodia (*n* = 4)	Egypt (*n* = 6)	Peru (*n* = 8)	Kenya (*n* = 6)
*bla*_CMY/LAT_	25%	nd *	nd	nd	nd
*bla*_LEN_	nd	nd	33%	nd	nd
*bla*_OXA-48_ family	nd	nd	nd	nd	17%
*bla*_PSE/CARB_	13%	nd	17%	nd	nd
*bla*_SHV_ family	nd	nd	50%	nd	nd
*bla*_TEM_ family	50%	50%	83%	25%	83%
*bla*_CTX-M-1_ family	nd	25%	17%	nd	nd
*bla*_CTX-M-9_ family	nd	nd	33%	nd	nd
*aac(3)-Id*	nd	nd	50%	nd	nd
*aac(3)-III*	nd	nd	nd	13%	nd
*aadA1/A2*	50%	nd	50%	75%	50%
*aadA7*	nd	nd	50%	nd	nd
*aphA1*	13%	nd	50%	63%	nd
*strA*	63%	75%	50%	25%	83%
*strB*	63%	75%	67%	25%	100%
*aphA6*	13%	nd	33%	nd	nd
*rmtD*	13%	nd	nd	nd	17%
*ere(A2)*	nd	nd	33%	nd	nd
*mph*(A)*/mph*(K)	nd	nd	17%	nd	17%
*tet*(A)	38%	50%	33%	88%	17%
*tet*(B)	50%	50%	nd	nd	17%
*tet*(C)	13%	nd	nd	nd	nd
*tet*(D)	nd	nd	17%	13%	nd
*tet*(G)	13%	nd	17%	nd	nd
*catA1/cat4*	25%	nd	nd	nd	33%
*floR*	25%	nd	17%	13%	17%
*cmr*	nd	nd	nd	nd	17%
*qnrS*	nd	25%	nd	nd	nd
*qacE*Δ*1*	38%	nd	83%	75%	50%
*sul1*	38%	nd	83%	75%	50%
*sul2*	50%	75%	33%	13%	100%
*sul3*	nd	nd	nd	13%	nd
*dfrA1*	nd	nd	nd	13%	33%
*dfrA7*	25%	nd	nd	nd	17%
*dfrA8*	nd	nd	nd	nd	17%
*dfrA12*	nd	nd	17%	13%	nd
*dfrA14*	nd	nd	nd	63%	50%
*dfrA19*	nd	nd	19%	nd	nd

* not detected.

**Table 6:** Percentage of *E. coli*, *Shigella* spp., and *Salmonella* isolates harboring at least one ARD to each of 11 classes of antimicrobial compounds.

Antimicrobial Class	Geographic Location				
	USA	Cambodia	Egypt	Peru	Kenya
β-Lactams	50.0	42.1	88.5	47.1	65.2
Aminoglycosides	77.3	52.6	100.0	88.2	95.7
Macrolides	54.5	31.6	80.8	52.9	47.8
Tetracyclines	95.4	94.7	92.3	88.3	70.0
Ansamycins	nd **	nd	nd	5.9	nd
Phenicols	81.8	63.2	80.8	64.7	47.8
Quinolones	nd	10.5	7.7	nd	4.3
Quaternary amines	22.7	nd	26.9	47.1	21.7
Streptothricins	13.6	10.5	42.3	1.8	17.4
Sulfonamides	63.6	47.4	73.1	82.4	95.7
Trimethoprim	31.8	26.3	69.2	70.6	78.3
Class 1 integron *	18.2	n/a ***	19.2	47.1	17.4
Class 2 integron *	9.1	n/a	34.7	5.9	13.0

* confirmed by *intI1*- and *intI2*-specific PCRs; ** not detected; *** not available for PCR confirmation.

## Supplementary Materials

Supplementary materials can be found at https://www.mdpi.com/1422-0067/21/16/5928/s1.

## Abbreviations

AG	Aminoglycoside
AMP	Antimicrobial peptide
AMR	Antimicrobial resistance
ANS	Ansamycins
ARD	Antimicrobial resistance determinant
ARDM	Antimicrobial Resistance Determinant Microarray
AZM	Azithromycin
BLA	β-Lactamase
CDC	Centers for Disease Control and Prevention
CLSI	Clinical and Laboratory Standards Institute
ESBL	Extended-spectrum β-lactamase
FQ	Fluoroquinolone
GEIS	Global Emerging Infections Surveillance and Response System
GLY	Glycopeptides
LIN	Lincosamides
MAC	Macrolides
MLS	Macrolide/lincosamide/streptogramin
MUP	Mupirocin
NAMRU	Naval Medical Research Unit
NRL	Naval Research Laboratory
NTS	Non-typhoidal *Salmonella*
PHE	Phenicols
PMQR	Plasmid-mediated quinolone resistance
PT	Platensimycin + platencin
QUA	Quaternary amines
SGI	*Salmonella* genomic island
SNP	Single nucleotide polymorphism
SRL	*Shigella* resistance island
STR	Streptothricin
SUL	Sulfonamides
SXT	Trimethoprim-sulfamethoxazole
TET	Tetracycline
TMP	Diaminopyrimidine/trimethoprim
USAMRD	US Army Medical Research Directorate, Africa/Kenya Microbiology Hub, Kericho
